# Vaccine-induced mucosal immunity to poliovirus: analysis of cohorts from an open-label, randomised controlled trial in Latin American infants

**DOI:** 10.1016/S1473-3099(16)30169-4

**Published:** 2016-12

**Authors:** Peter F Wright, Ruth I Connor, Wendy F Wieland-Alter, Anne G Hoen, Austin W Boesch, Margaret E Ackerman, M Steven Oberste, Chris Gast, Elizabeth B Brickley, Edwin J Asturias, Ricardo Rüttimann, Ananda S Bandyopadhyay

**Affiliations:** a Department of Pediatrics, Dartmouth College, Hanover, NH, USA; b Department of Microbiology and Immunology, Dartmouth College, Hanover, NH, USA; c Department of Epidemiology, Geisel School of Medicine, Dartmouth College, Hanover, NH, USA; d Thayer School of Engineering, Dartmouth College, Hanover, NH, USA; e Centers for Disease Control and Prevention, Atlanta, GA, USA; f Fred Hutchinson Cancer Research Center, Seattle, WA, USA; g Departments of Pediatrics and Epidemiology, University of Colorado Denver School of Medicine, Aurora, CO, USA; h Fighting Infectious Diseases in Emerging Countries (FIDEC), Miami, FL, USA; i Bill & Melinda Gates Foundation, Seattle, WA, USA

## Abstract

**Background:**

Identification of mechanisms that limit poliovirus replication is crucial for informing decisions aimed at global polio eradication. Studies of mucosal immunity induced by oral poliovirus (OPV) or inactivated poliovirus (IPV) vaccines and mixed schedules thereof will determine the effectiveness of different vaccine strategies to block virus shedding. We used samples from a clinical trial of different vaccination schedules to measure intestinal immunity as judged by neutralisation of virus and virus-specific IgA in stools.

**Methods:**

In the FIDEC trial, Latin American infants were randomly assigned to nine groups to assess the efficacy of two schedules of bivalent OPV (bOPV) and IPV and challenge with monovalent type 2 OPV, and stools samples were collected. We selected three groups of particular interest—the bOPV control group (serotypes 1 and 3 at 6, 10, and 14 weeks), the trivalent attenuated OPV (tOPV) control group (tOPV at 6, 10, and 14 weeks), and the bOPV–IPV group (bOPV at 6, 10, and 14 weeks plus IPV at 14 weeks). Neutralising activity and poliovirus type-specific IgA were measured in stool after a monovalent OPV type 2 challenge at 18 weeks of age. Mucosal immunity was measured by in-vitro neutralisation of a type 2 polio pseudovirus (PV2). Neutralisation titres and total and poliovirus-type-specific IgG and IgA concentrations in stools were assessed in samples collected before challenge and 2 weeks after challenge from all participants.

**Findings:**

210 infants from Guatemala and Dominican Republic were included in this analysis. Of 38 infants tested for mucosal antibody in the tOPV group, two were shedding virus 1 week after challenge, compared with 59 of 85 infants receiving bOPV (p<0·0001) and 53 of 87 infants receiving bOPV–IPV (p<0·0001). Mucosal type 2 neutralisation and type-specific IgA were noted primarily in response to tOPV. An inverse correlation was noted between virus shedding and both serum type 2 neutralisation at challenge (p<0·0001) and mucosal type 2 neutralisation at challenge (p<0·0001).

**Interpretation:**

Mucosal type-2-specific antibodies can be measured in stool and develop in response to receipt of OPV type 2 either in the primary vaccine series or at challenge. These mucosal antibodies influence the amount of virus that is shed in an established infection.

**Funding:**

Bill & Melinda Gates Foundation.

## Introduction

As the spread of poliovirus is progressively constrained both geographically and in circulating lineages, crucial decisions are being made about future vaccination strategies to achieve and sustain the final eradication of poliomyelitis.^1^, ^2^ These plans include elimination of the type 2 component of the trivalent oral attenuated poliovirus vaccine (tOPV), as recommended by the Strategic Advisory Group of Experts on Immunization, and replacement with bivalent oral poliovirus vaccine (bOPV) accompanied by the introduction of at least one dose of inactivated polio vaccine (IPV) into the regimen, enhanced environmental surveillance, and the eventual elimination of any use of the live oral poliovirus vaccines (OPVs) in immunisation programmes. The cessation of the use of OPV type 2 and the substitution of at least one dose of IPV occurred as planned globally in April and May, 2016.

These recommendations, made in 2012, were based on expert opinion and the epidemiological situation of poliovirus transmission, but no immunogenicity and safety data for bOPV–IPV routine immunisation schedules compared with tOPV immunisation schedules—particularly the effects on intestinal immunity against type 2 Sabin virus—were available to inform them. The IPV and OPV both prevent paralytic poliomyelits in the vaccine recipient, and both have interrupted transmission, although the impact of IPV on transmission has largely been reported in developed countries. Mucosal immunity is presumed to have a key role in limitation of enteric and pharyngeal infection with poliovirus, and hence could be crucial in diminishing the efficiency of transmission. Yet the notion that IPV will be central to maintenance of eradication is based on the potential development of vaccine-associated paralytic polio in OPV recipients,^3^ chronic OPV infection in immunodeficient vaccine recipients,^4^ and circulation of vaccine-derived polioviruses.^5^

**Research in context**

**Evidence before this study**

We searched PubMed with the term “polio mucosal immunity” for articles published in English between Jan 31, 1975, and Dec 31,2015. We identified several studies in which virus shedding after oral challenge was used as a surrogate for mucosal immunity, but very few in which investigators attempted to measure IgA specific antibody in the stool and none in which neutralising antibody in the stool was successfully measure and correlated with virus recovery on oral poliovirus challenge.

**Added value of this study**

We present the first data showing the impact of trivalent attenuated oral poliovirus vaccine (tOPV), bivalent oral poliovirus vaccine (bOPV), and a combination of bOPV and inactivated poliovirus vaccine (IPV) on intestinal immunity against type 2 poliovirus when administered according to the Expanded Program on Immunization schedule and document the effect of that immunity on subsequent challenge with the proposed outbreak response tool of the future, monovalent oral poliovirus vaccine (mOPV) type 2. Immunity is measured by restriction of virus shedding, neutralisation of virus in the stool, and mOPV type 2 IgA specific antibody. The correlations of each with the other strengthens the concept that we now have tools to measure mucosal immunity and predict the effectiveness of a vaccine regimen in limiting outbreaks as we approach polio eradication.

**Implications of the available evidence**

Our novel data have direct and immediate global policy implications and point to the unique ability of tOPV to stimulate mucosal immunity, the dissociation of serum and mucosal antibody responses, and the crucial role of mucosal immunity in preventing infection on an mOPV type 2 challenge. They provide support for the global withdrawal of type-2-containing oral poliovirus vaccines to eliminate the only source of paralytic disease caused by this serotype, and show that, although IPV is highly protective against paralytic disease in the individual vaccinee, it has only a small effect on subsequent virus shedding and thus could allow a continued train of transmission. Our data will help national, regional, and global policy makers to develop public health strategies to achieve and sustain polio eradication in the near term and long term.

The comparative ability of OPV and IPV to induce mucosal immunity has been explored since the early days of polio research. Ouchterlony double diffusion plates were used to show that OPV induces enteric IgA, whereas IPV does not, unless preceded by OPV.^6^, ^7^ The concept that mucosal immunity was dependent on previous vaccination with OPV was supported by challenging IPV and OPV recipients with OPV.^8^, ^9^ Combination of OPV and IPV to broaden immunogenicity was introduced 18 years ago.^10^ More recently, investigators in Cuba,^11^ India,^12^, ^13^ Bangladesh,^14^ Chile,^15^ and four Latin American countries in the Fighting Infectious Diseases in Emerging Countries (FIDEC)^16^ consortium have used challenge with either monovalent OPV (mOPV) or tOPV as a measure of the immunogenicity and protection afforded by previous immunisation with OPV or IPV, either individually or in combination.

Anticipatory studies of the mechanisms that limit poliovirus replication are crucial to inform decisions by the Global Polio Eradication Initiative to move from OPV to IPV. Studies of mucosal immunity induced by OPV, IPV, and mixed schedules will define the best strategy, not only for prevention of paralytic disease, but also for the true eradication of all circulating poliovirus. The lack of definitive information in this area was shown in 2013 by leaders in the field, who expressed very diverse views on the effectiveness of IPV in limiting virus replication at mucosal sites and thus preventing transmission.^17^ Previous studies have yet to link mucosal responses measured by current immunological techniques to the type of vaccine given or to virus shedding on challenge. To address this gap in knowledge, we measured polio-specific mucosal IgA responses and virus-neutralising capacity of stool samples and correlated these findings with vaccine regimen and virus shedding on OPV challenge.

## Methods

### Study design and participants

The FIDEC study (NCT 01831050) was designed to explore the safety, immunogenicity, and protective efficacy of combinations of IPV and OPV in anticipation of the elimination of type 2 OPV from global vaccination use. Infants in the study were randomly assigned to nine groups to assess the efficacy of two schedules of bOPV and IPV and challenge with monovalent type 2 OPV. Stool samples were collected during the FIDEC trial as reported^16^ and provided to us so that we could analyse mucosal responses. Because of the large number of samples collected and the complexity of the analyses, stool specimens were specifically selected from three non-overlapping groups of particular scientific interest: the bOPV control group, in which 210 infants received bOPV (serotypes 1 and 3) at 6, 10, and 14 weeks, followed by challenge with mOPV type 2 at 18 weeks; the tOPV control group, in which 100 infants received tOPV at 6, 10, and 14 weeks, with mOPV type 2 challenge at 18 weeks; and the bOPV–IPV group, in which 210 infants received bOPV at 6, 10, and 14 weeks and one dose of the IPV at 14 weeks, with mOPV type 2 challenge at 18 weeks. Other groups in the parent study that were not analysed in ours received either two doses of IPV in addition to OPV or were challenged at 40 weeks rather than 18 weeks with mOPV type 2, or both.^16^

The study was approved by the Dartmouth College Committee for the Protection of Human Subjects and by the FIDEC ethical review process. Provision had been made in the initial consent for future studies and it was judged unnecessary to reconsent the volunteers.

### Procedures

The FIDEC study was implemented in four countries (Colombia, Dominican Republic, Guatemala, and Panama). We determined that analysis of all samples from two of these countries—Dominican Republic and Guatemala—would yield statistically meaningful data for our study. Because limited inter-country differences were noted, results from the two countries were pooled for all data analyses.

Stool samples (5–10 g) were collected and frozen in aliquots in the country of origin. Samples were then shipped frozen to the Geisel School of Medicine at Dartmouth (Hanover, NH, USA). Assays quantifying virus neutralisation titres and IgA concentrations in stools were done at Dartmouth as previously reported.^18^, ^19^ Mucosal immunity was measured by in-vitro neutralisation of a type 2 polio pseudovirus (PV2). Neutralisation titres and total and polio-type-specific IgG and IgA concentrations in stools were assessed in samples collected before challenge and 2 weeks after challenge from all subjects. The same measurements were done on samples collected weekly for the first 4 weeks after challenge from 49 randomly chosen participants by the Dartmouth team (before any knowledge of group allocation). Serum neutralisation titres before challenge and a week later, and virus shedding weekly after challenge, were measured at the Polio and Picornavirus Laboratory Branch of the Centers for Disease Control and Prevention (Atlanta, GA, USA). Vaccine group assignment, serum neutralisation titres, and virus shedding were unblinded to the Dartmouth study team after completion of sample testing and data sharing.

### Statistical analyses

Differences in the proportions of children with virus shedding and with measurable PV2 mucosal neutralisation were compared across the three vaccine regimen groups with Pearson's χ^2^ test. We used ANOVA to compare the geometric means of observed virus shedding, stool and serum neutralisation titres, and mucosal IgA concentrations across the three groups. Differences between pairs of groups and between visits within each group were compared with Tukey's honest significant difference method—a simultaneous comparison of all pairwise means that identifies differences between two means that are greater than the expected SE and is robust when sample sizes are unequal between groups. Differences were deemed significant and p values reported when p was less than 0·05.

We used Spearman's rank correlation coefficients to examine correlations between shedding 1 week after type 2 mOPV challenge and poliovirus-type-specific serum and stool neutralisation titres and mucosal IgA concentrations at the time of challenge. We plotted the median (IQR) concentrations of each y variable against the mean for each third of the x variables, which allowed us to assess the shape of any correlation without assuming linearity a priori.

### Role of the funding source

ASB is an employee of the study funder, and was involved in study design, data interpretation, and writing of the report. The funder had no role in data collection. All authors had full access to all the data in the study and share final responsibility for the decision to submit for publication.

## Results

**Figure 1:**
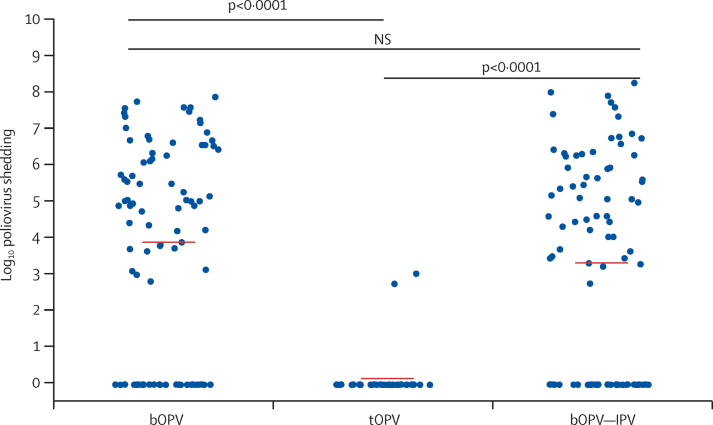
Poliovirus shedding in stools 1 week after mOPV type 2 challenge p<0·0001 after bOPV, tOPV, or bOPV–IPV at 6, 10, and 14 weeks. mOPV type 2 challenge was at 18 weeks. mOPV=monovalent oral poliovirus vaccine. bOPV=bivalent oral poliovirus vaccine. tOPV=trivalent attenuated oral poliovirus vaccine. IPV=inactivated poliovirus vaccine. NS=not significant.

In total, 586 samples were analysed from 210 infants given bOPV (n=85), tOPV (n=38), or bOPV–IPV (n=87), which represented approximately 40% of the children in each group. Virus shedding was diminished 1 week after mOPV type 2 challenge by previous receipt of a vaccine containing homologous live type 2 in the immunisation schedule (figure 1). Infants in the tOPV group demonstrated very limited virus shedding (two [5%] of 38 infants) compared with infants receiving bOPV (59 [69%] of 85) or bOPV–IPV (53 [61%] of 87; χ^2^ [1, n=123]=43·2, p<0·0001 for tOPV group *vs* bOPV group; χ^2^ [1, N=125]=33·3, p<0·0001 for tOPV group *vs* bOPV–IPV group). The full pattern of shedding is reported separately.^16^

**Figure 2:**
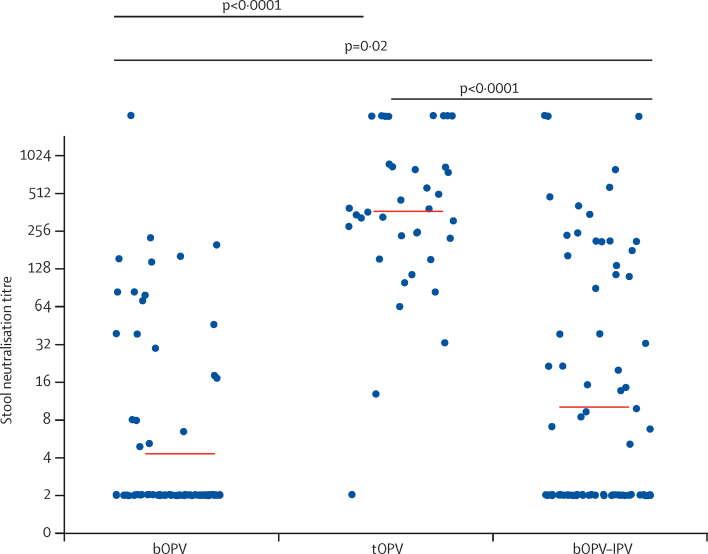
Type 2 polio pseudovirus neutralising titre measured in stools at the time of mOPV type 2 challenge p<0·0001 after bOPV, tOPV or bOPV–IPV at 6, 10, and 14 weeks. mOPV type 2 challenge was at 18 weeks. mOPV=monovalent oral poliovirus vaccine. bOPV=bivalent oral poliovirus vaccine. tOPV=trivalent attenuated oral poliovirus vaccine. IPV=inactivated poliovirus vaccine.

Mucosal immunity at the time of the mOPV type 2 challenge was significantly associated with previous receipt of a vaccine containing the OPV type 2 component—ie, tOPV (figure 2). 14 (17%) of 82 individuals given bOPV also demonstrated mucosal PV2 neutralisation. One dose of IPV at 14 weeks (ie, in the bOPV–IPV group) was associated with measurable PV2 mucosal neutralisation in 21 (26%) of 82 infants, compared with 35 (42%) of 84 infants given bOPV (χ^2^ [1, N=166]=4·79, p=0·03; figure 2). However, the additional dose of IPV had no significant effect on the level of virus shedding compared with bOPV only (figure 1). At the time of mOPV type 2 challenge, type 1 polio pseudovirus neutralisation titres were similar in the three groups (data not shown).

**Figure 3:**
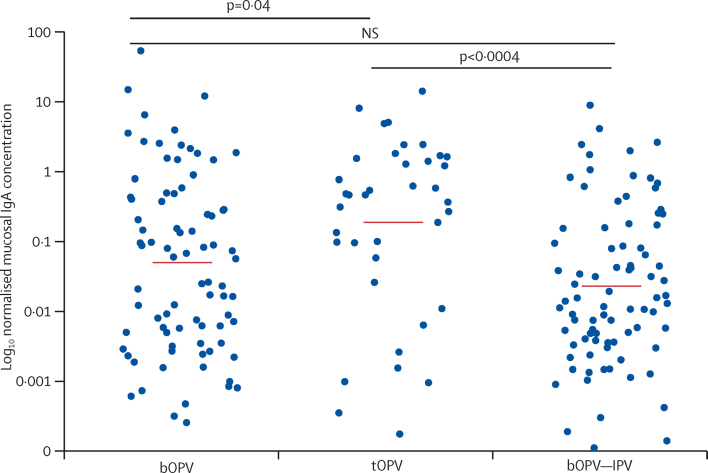
IgA antibody concentrations measured in stools by Luminex assay after mOPV type 2 challenge Normalised IgA concentration is the normalised polio type 2 specific IgA in stool relative to a total IgA serum standard IgA. p=0·0007 after bOPV, tOPV or bOPV–IPV at 6, 10, and 14 weeks. mOPV type 2 challenge was at 18 weeks. mOPV=monovalent oral poliovirus vaccine. bOPV=bivalent oral poliovirus vaccine. tOPV=trivalent attenuated oral poliovirus vaccine. IPV=inactivated poliovirus vaccine.

**Figure 4:**
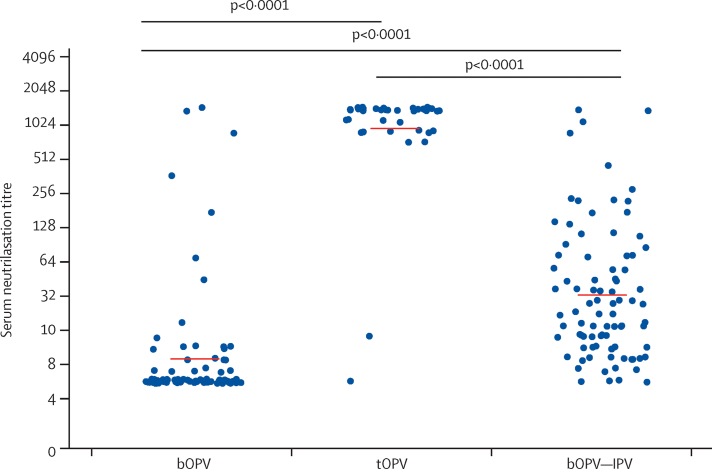
Type-2-specific neutralising titre in serum after mOPV type 2 challenge p<0·0001 after bOPV, tOPV, or bOPV–IPV at 6, 10, and 14 weeks. mOPV type 2 challenge was at 18 weeks. mOPV=monovalent oral poliovirus vaccine. bOPV=bivalent oral poliovirus vaccine. tOPV=trivalent attenuated oral poliovirus vaccine. IPV=inactivated poliovirus vaccine.

Type-2-specific IgA concentrations measured in stool at the time of mOPV type 2 challenge were significantly higher in infants given tOPV than in those receiving bOPV or bOPV–IPV (figure 3). Total IgG concentrations in the stool specimens were much lower than those of IgA; polio-strain-specific IgG antibodies were rarely detected (data not shown). Type-2-specific serum neutralisation titres were significantly higher in infants who received a homologous type 2 vaccine (ie, those in the tOPV and bOPV–IPV groups) than in those who did not (figure 4).

**Figure 5:**
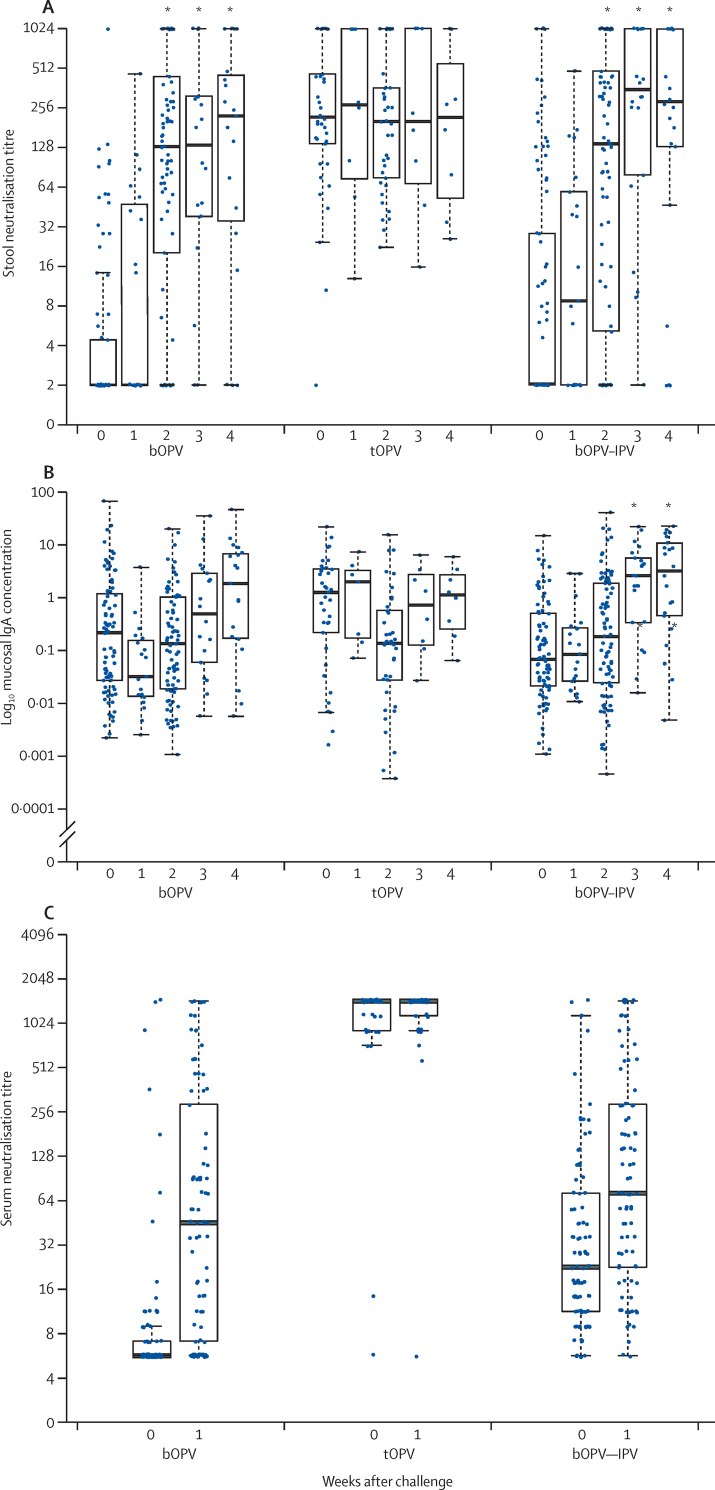
Mean PV2-specific neutralisation titres in stool (A), mean concentrations of PV2-specific mucosal IgA in stool (B), and mean type-2-specific serum neutralisation titres (C) at the time of mOPV type 2 challenge and weekly thereafter Mucosal IgA concentration is mucosal type 2 specific IgA in stool relative to a total IgA serum standard. PV2=type 2 polio pseudovirus. mOPV=monovalent oral poliovirus vaccine. bOPV=bivalent oral poliovirus vaccine. tOPV=trivalent attenuated oral poliovirus vaccine. IPV=inactivated poliovirus vaccine. *Indicates a significant increase in titre compared with that at the time of challenge.

Mucosal neutralising activity to PV2 developed rapidly after administration of the mOPV type 2 challenge in the bOPV and bOPV–IPV groups, reaching the levels of the tOPV group by 2 weeks after challenge (figure 5A). Mean IgA concentrations also increased in stool by 3–4 weeks after challenge in the bOPV and bOPV–IPV groups (figure 5B). By comparison, the vaccine regimen did not affect mucosal type-1-specific or type-3-specific IgA concentrations at the time of challenge, and these concentrations did not rise significantly after mOPV type 2 challenge (data not shown).

Serum was obtained immediately before challenge and 1 week later under the assumption that this practice would capture a rapid anamnestic antibody response to vaccine priming. Serum neutralisation titres determined for the two available timepoints showed no significant increase 1 week after challenge in the bOPV and bOPV–IPV groups (figure 5C). A comparison of both the mucosal and serum neutralisation titres 1 week after mOPV type 2 challenge showed equivalent responses in the bOPV and bOPV–IPV groups (figure 5A, 5C).

Virus shedding was restricted 1 week after mOPV type 2 challenge in the tOPV group, which had received OPV type 2 as a component of the trivalent vaccine, compared with that in the bOPV and bOPV–IPV groups, neither of which had received OPV type 2 (figure 1; p<0·0001 for both). Similarly, significantly higher mucosal neutralising responses to PV2 were noted in tOPV recipients at the time of mOVP type 2 challenge than were noted in those in the bOPV and bOPV–IPV groups (figure 2).

**Figure 6:**
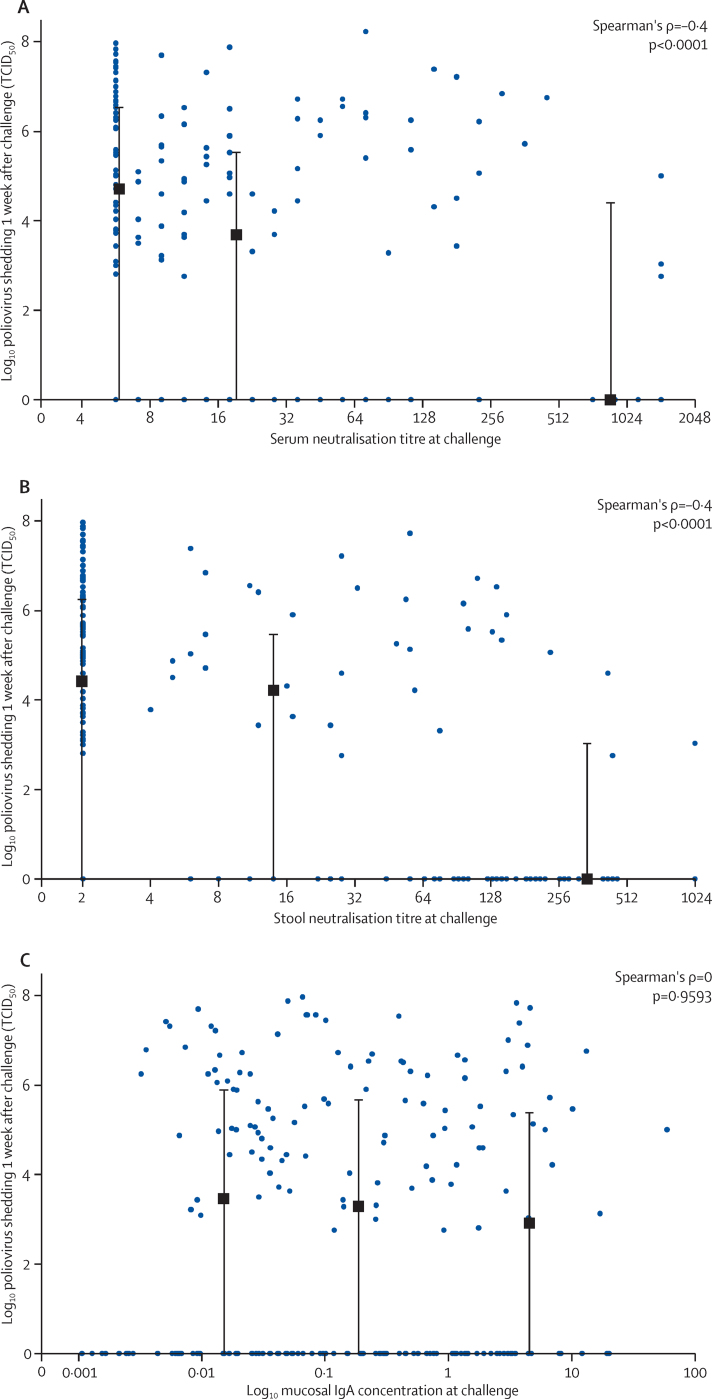
Relation between peak type-2-specific neutralisation titres in serum (A), peak type-2-specific neutralisation titres in stool (B), and type-2-specific mucosal IgA concentrations (C) at the time of mOPV type 2 challenge, and virus shedding 1 week later Data in each panel represent combined responses for all three vaccine groups. Black squares and bars indicate the median (IQR) of virus shedding within thirds of antibody responses plotted against the mean of the antibody responses within each third. mOPV=monovalent oral poliovirus vaccine. TCID_50_=50% tissue culture infective dose.

Separate analyses of the distribution of the type-2-specific serum and stool neutralisation responses at the time of mOPV type 2 challenge demonstrated a significant relation between the heights of each response with the subsequent reduction in virus shedding 1 week after challenge (figures 6A, 6B). Concentrations of type-2-specific IgA measured at challenge did not correlate with subsequent virus shedding 1 week later (figure 6C).

**Figure 7:**
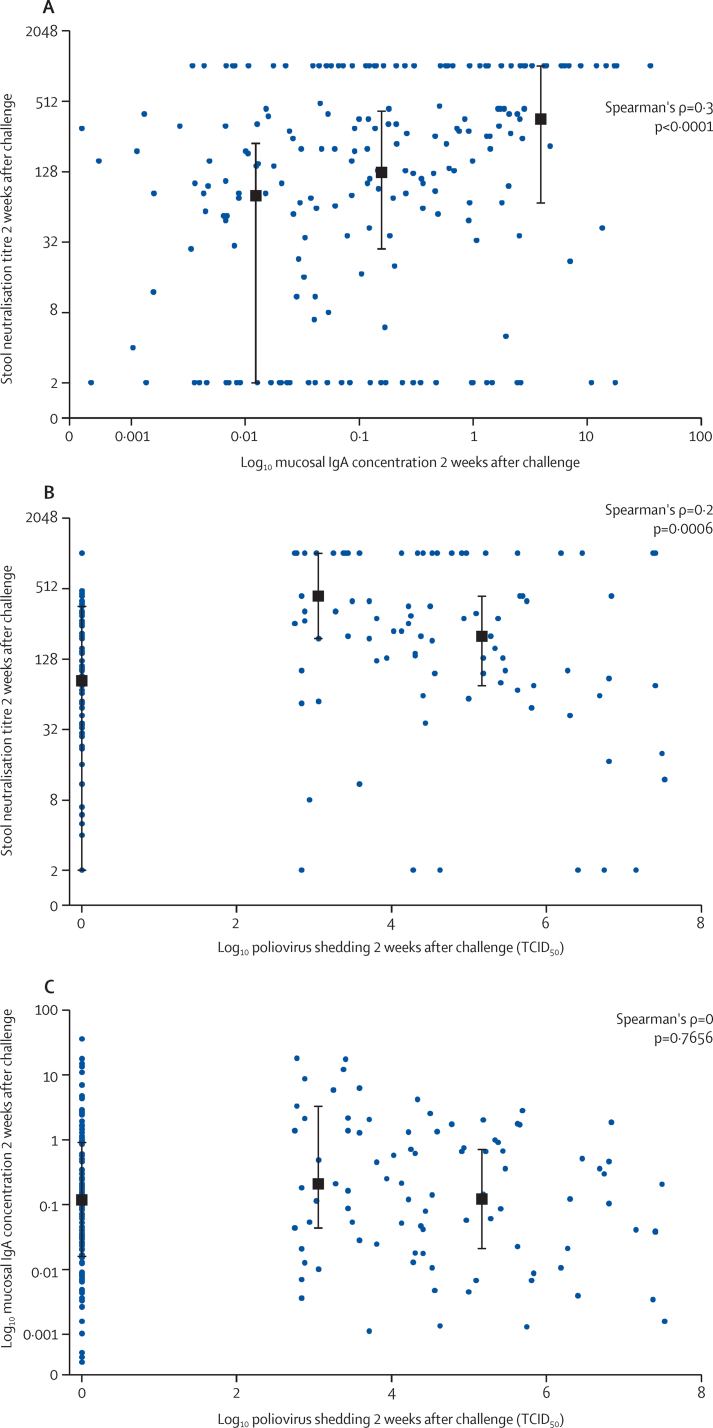
Relation between peak stool neutralisation titre and mucosal IgA concentrations (A), peak stool neutralisation titre and poliovirus shedding (B), and mucosal IgA concentrations and poliovirus shedding (C) 2 weeks after mOPV2 challenge among individuals shedding virus Data in each panel represents combined responses for all three vaccine groups. Black squares and bars indicate the median (IQR) of the y variables within thirds of x variables plotted against the mean of the x variables within each third. mOPV=monovalent oral poliovirus vaccine. TCID_50_=50% tissue culture infective dose.

A comparison of PV2-specific mucosal neutralisation titres with IgA concentrations in stools from the tOPV recipients at the time of challenge was significant (p=0·043), with a weak correlation (r=0·33). Notably, by 2 weeks after mOPV type 2 challenge—when neutralising PV2-specific antibody responses in stool were significantly increased in the bOPV and bOPV–IPV groups compared with those at time of challenge (figure 5A)—the correlation of mucosal neutralisation with type-2-specific IgA concentrations in stool was strong (figure 7B).

In infants shedding virus after mOPV type 2 challenge, we noted a significant inverse correlation between the height of the mucosal response, as measured by both virus neutralising activity and IgA concentrations in stool, and the amount of virus concurrently shed 2 weeks after challenge (figure 7B). A correlation of virus shedding with mucosal IgA was not noted (figure 7C).

## Discussion

Our results suggest that mucosal neutralising type-2-specific antibody, generated by previous serotype-specific OPV exposure, highly corresponds with resistance to challenge with mOPV type 2. Although type-2-specific serum neutralising antibody also influenced the initial replication of mOPV type 2 after challenge, further amplification of this response by inclusion of a dose of IPV in the immunisation regimen (ie, in the bOPV–IPV group) did not further reduce virus recovery. Restriction of virus shedding after mOPV type 2 challenge was linked to previous receipt of homotypic OPV (tOPV). Additionally, little if any immunity to type 2 mOPV was induced by receipt of heterotypic bOPV. Although unlikely, resistance to mOPV type 2 challenge could be due to induction of some other type of protective immunity unrelated or additional to the observed mucosal and serum neutralisation responses. Because the number of infants in the tOPV group who shed virus after challenge was quite small, we were unable to rigorously implicate mucosal antibody as the key protective factor within that group. In general, discerning the relative contribution of mucosal or serum neutralisation responses to the recorded restriction of virus shedding was complicated by the limited number of infants shedding virus after immunisation with tOPV; confounding serum and mucosal antibody responses that were not attributable to vaccine receipt in the trial and were noted in infants from all groups; and a general concordance of the mucosal and serum neutralising responses after immunisation with tOPV.

Furthermore, we conclude that serum neutralising antibody, the traditional measure of vaccine-induced immunity, is a limited determinant of virus replication in the intestinal tract. This observation is crucial to decisions about future approaches to elimination of poliovirus circulation and sustainment of a polio-free world. The experience in Israel, where sustained circulation of wild-type polio occurred in a population highly immunised with IPV,^20^ provides further evidence of the importance of adopting an endgame vaccine regimen for the interim that also induces mucosal immunity.

Together with our preceding study,^18^ in this study we establish a unique method to measure strain-specific mucosal antibody responses to poliovirus that reveals functionality (virus neutralisation) at very high titres (often ≥512) equivalent to those measured in serum. Previous attempts to measure mucosal immunity have largely focused on measurements of binding antibody, primarily of the IgA class. Luminex provides a state-of-the-art platform for polio-specific IgA determination. Substantial difficulties have also been encountered in development of reproducible assays to assess virus neutralisation mediated by respiratory, genital, or enteric specimens. Aside from the recognised complications of sample variability during collection, processing, and assaying, measurement of functional activity in biological assays is fraught with challenges associated with cellular toxicity or cell culture contamination by the specimen, or both. Most of these shortcomings are subverted by the rapid readout of the polio pseudovirus assay, which provides a sensitive and reproducible means of assessing neutralisation after a single cycle of virus replication.^18^

Several questions remain unanswered. First, what is the origin of the serum and mucosal neutralising responses seen in the bOPV group before OPV type 2 challenge, which theoretically had not yet received any type 2 vaccine? Serum responses could indicate residual maternal antibody or antibody acquired during intercurrent infection, although efforts were made to avoid any secondary exposure to vaccine virus in the family setting. Mucosal antibody responses might also reflect variation in sample collection, storage, or residual maternal breast milk antibody. Second, what mediates the early neutralising response noted on challenge? IgA is assumed to be the major mediator of mucosal immunity.^21^ If so, why was there not a better correlation between the height of mucosal neutralisation and IgA concentrations at challenge and the amount of virus shedding 1 week later? In determining virus titre, the samples were frozen on collection and treated with chloroform immediately after thawing. We have shown that chloroform rapidly inactivates antibody. Thus neutralisation has little opportunity to occur in the limited timeframe between evacuation and sample processing. With respiratory syncytial virus, mucosal antibody was found bound to cell-associated virus in secretions and only later were investigators able to detect free antibody.^22^ The poorly defined site or sites of replication of poliovirus in the enteric tract (eg, Peyer's patches) could make the virus inaccessible to antibody secreted into the intestinal lumen.^23^ Furthermore, we would conclude that the induction of immunity noted 1 week after challenge is not necessarily indicative of priming, because it was reported in children who were undergoing their initial exposure to type 2 vaccine. Finally, the duration of mucosal immunity is unknown, but we suspect it to be shorter than that of serum antibody, and thus the protection recorded almost certainly will wane.^24^ In this study, we could not address duration or the concept that previous OPV mucosal immunity is boosted by IPV.^25^

In summary, another tool has been added to assessments of immunity after receipt of polio vaccines. Our findings move beyond the initial observation that OPV is a much better mucosal immunogen than IPV, as evidenced by greater inhibition of virus replication upon challenge, and show a mechanism by which this anti-viral activity might occur. In defining a mechanism for measuring mucosal immunity for polio, there is a mandate to extend these observations and apply these findings to other live virus vaccines that are either in clinical use (eg, influenza, rotavirus) or actively under development (eg, respiratory syncytial virus).
